# Online medical education using a Facebook peer-to-peer learning platform during the COVID-19 pandemic: a qualitative study exploring learner and tutor acceptability of Facebook as a learning platform

**DOI:** 10.1186/s12909-023-04268-3

**Published:** 2023-05-01

**Authors:** Joshua Chambers, Khaylen Mistry, Joel Spink, Jordan Tsigarides, Pauline Bryant

**Affiliations:** 1 Norwich Medical School, University of East Anglia, Norwich, UK; 2 Norfolk and Norwich University Hospital, Norwich, UK

**Keywords:** e-learning, Professionalism, Education environment, Collaborative/peer-to-peer, Medical education research

## Abstract

**Background:**

In recent years, higher education institutions have been moving teaching online, accelerated by the pandemic. The Remote Learning Project (RLP), based at the Norwich Medical School (NMS) in the United Kingdom (U.K.), was a peer-to-peer teaching program developed to supplement medical school teaching during the pandemic. The teaching was delivered through Facebook using peer-to-peer teaching. Tutors were final year medical students, teaching medical student learners in lower years. Tutors and learners perception of peer-to-peer online learning delivered through the Facebook Social Media (SoMe) platform was investigated.

**Methods:**

This qualitative study recruited tutor and learner participants from NMS by email, participation in the study was voluntary. Online semi-structured interviews of both tutors and learners in the remote learning project were conducted. The data was analysed using thematic analysis.

**Results:**

Seven participants were interviewed. Five themes were identified; education (learning/teaching), productivity, data security, professionalism, and usability of the platform. Learners enjoyed the asynchronous nature of the platform and both learners and tutors enjoyed the peer-to-peer nature of the RLP, including the ability to immediately and easily answer on Facebook comments. Some learners felt distracted on Facebook, whilst others enjoyed the reminders. The mix of social and professional on the platform was met with caution from tutors. Both learners and tutors enjoyed the familiarity of the platform.

**Conclusions:**

The study found that SoMe may be a credible platform to deliver online peer-to-peer teaching. Educators should consider the ergonomics of SoMe platforms when designing online curriculums. Guidelines for educators should be developed to better guide educators on the effective and safe use of SoMe as a learning tool.

**Supplementary Information:**

The online version contains supplementary material available at 10.1186/s12909-023-04268-3.

## Introduction

In recent years the delivery of undergraduate medical education has begun to include online elements. This was accelerated during the COVID-19 pandemic when it was necessary to rapidly convert large amounts of teaching to an online platform. The pandemic brought about significant challenges, but also highlighted new ways of doing things which brings exciting opportunities [1]. Higher education institutions are becoming increasingly reliant on technology in education, using learning management systems to coordinate curriculums and deliver teaching. Online teaching is diverse and flexible and there is much to learn about the best way to use it both as an educator and a learner.

A systematic review that explored knowledge, skills, satisfaction and outcomes found that online learning has equivalent efficacy to traditional teaching [1] but, there are still many unanswered questions about its use. A range of online teaching methods exist such as live or recorded lectures, videos, discussion forums and massive open online courses (MOOCs). A survey of medical students showed that a social media (SoMe) platform is used by 98% of medical students but concluded that only a minority used it for academic study [2].

Of all SoMe platforms, Facebook has been shown to be the most frequently visited by higher education scholars [3]. Its familiarity amongst students, combined with its interactivity and effectiveness, make it a possible learning environment. As it is online, it can also be used for asynchronous learning and is easily accessible on computers, tablets and mobile phones. Medical students use Facebook for communication, mentoring, collaboration, student wellbeing and blended learning. A unique feature of Facebook is the social aspect, which may distinguish it from other teaching methods and facilitate learning via social mechanisms. This fits in with the digital learning theory of connectivism, allowing increased learning and motivation through digital networks [4]. Facebook is received well by medical students, being used to share learning materials and case study discussions [5]. Nicolai et al. found that medical students used Facebook to organise study and discuss topics, determining it to be an essential complement to their medical school curriculum [6]. Ali et al. reported that medical students used Facebook for collaborative learning in asking questions and discussing work. In addition, it was used for revision by sharing study resources, and sharing experiences [7].

A limitation of existing literature is the lack of research on Facebook being used as a dedicated teaching platform. Most research focuses on generic Facebook use alongside main curriculum teaching methods.

Research highlights cultural resistance to Facebook, due to perceived risk of distractions and unfamiliarity with Facebook as a teaching platform [8–10]. Teachers’ perception of Facebook remains ambivalent [11]. Over the past decade Facebook has expanded to become increasingly integrated for education in other settings outside medicine, corporate and business purposes [12, 13]. Most literature evaluating Facebook as a teaching platform does not explore tutor perceptions [14]. There is paucity of data on the acceptability of Facebook in medical education and our qualitative research study aims to add to this knowledge.

Peer-to-peer learning involves learners teaching other learners and is an effective teaching method [15]. It has a high student satisfaction who perceive it to improve their learning and confidence [16]. It fits into a constructivist approach to medical education theory, with students perhaps feeling more able to interact with and learn with peers when compared to faculty [17] and draws on ideas of scaffolding and the Zone of Proximal Development that focuses on the idea that students can learn from those just ahead of them [18].

**Fig. 1:**
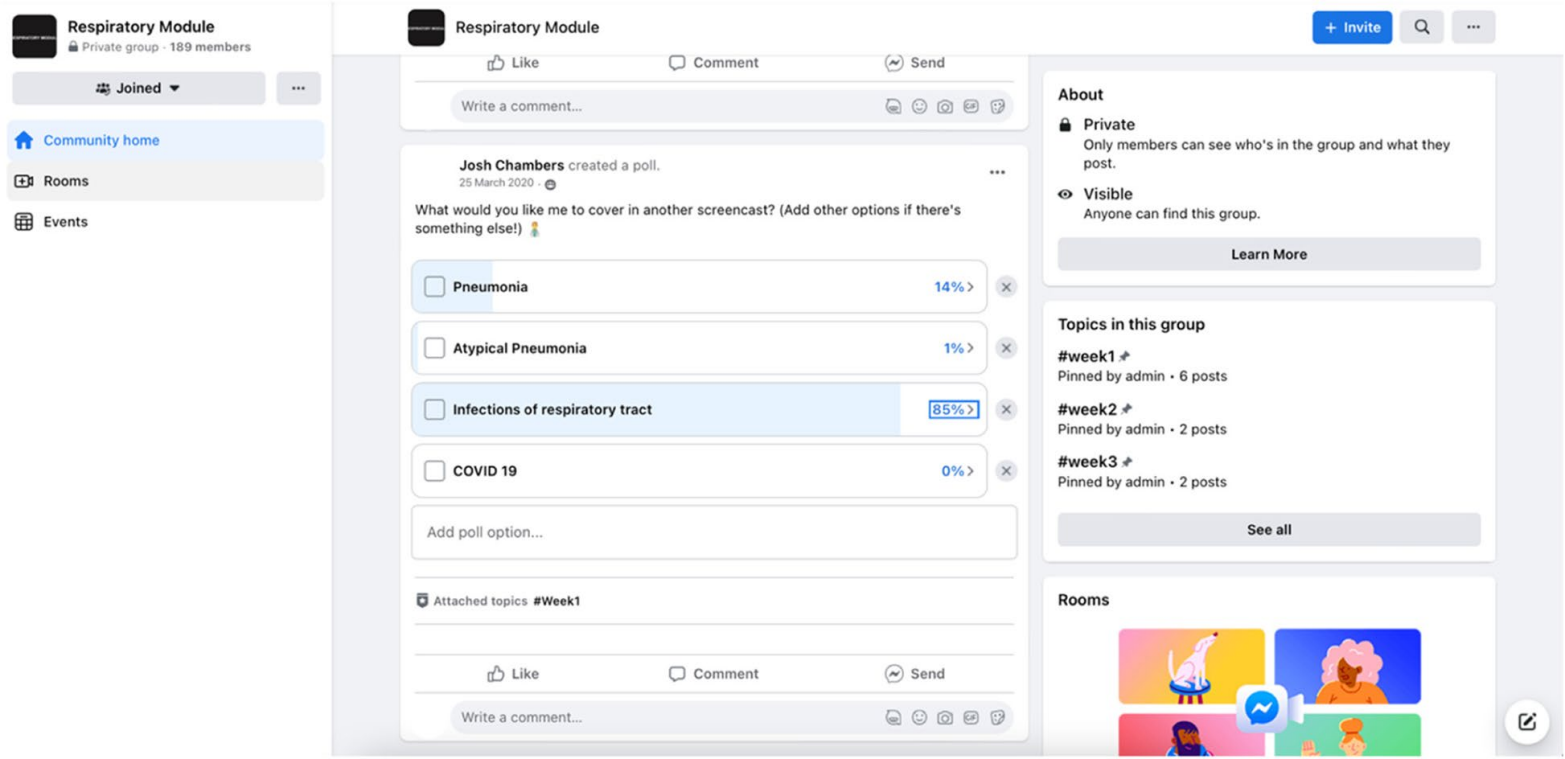
Screenshot from the Remote Learning Project closed Facebook group

Norwich Medical School (NMS) has an integrated curriculum and students complete 15 modules over 5 years consisting of lectures, seminars, clinical placements, and problem-based learning. In 2021, during the first COVID lockdown, all face-to-face teaching was cancelled. Following this, a Remote Learning Project (RLP) was formed with the aim to supplement medical school teaching as many clinical tutors at the medical school were unable to deliver teaching due to clinical pressures. The RLP was created and run by a group of 4 final year medical students and 1 intercalating medical student (Project Leaders) being overseen by a senior faculty member (PB). The final year medical students had completed final examinations early due to the pandemic, but were awaiting results. They are referred to here as tutors. The RLP aimed to deliver supplementary teaching to years 2–4 of the medical school via peer-to-peer learning using Facebook. These medical students will be referred to as the learners. 52 volunteer tutors were recruited by the Project Leaders to deliver the teaching. Multiple teaching methods were included such as PowerPoints, screencasts, live Q&A sessions and written notes. All were delivered via Facebook. The Facebook group was accessible to year 2–4 medical student learners with approximately 170 students in each year group (Fig. 1). Admittance was regulated by the tutors in a closed Facebook group. Both synchronous and asynchronous content was uploaded each week for 9 modules over a 4-month period. An average of 1–6 h of teaching was delivered per module per week. Student participation in the RLP was voluntary.

This qualitative study aimed to investigate the acceptability for student and tutors of the online learning Facebook platform. The hypothesis is that Facebook might be a useful peer-to-peer learning platform. The Objective is to understand the advantages and disadvantages of Facebook as a learning platform.

## Methods

Ethical approval (2020/21–030) was obtained from the University of East Anglia’s Faculty of Medicine and Health Sciences ethics committee.

### Participants

All participants in the study were students at the University of East Anglia in March 2020 who had taken part in the Remote Learning Project (RLP) on Facebook. There were two groups of participants in this study. “Learners” were medical students in years 2–4 of the Bachelor of Medicine, Bachelor of Surgery (MBBS) undergraduate curriculum in March 2020 who used the RLP as a teaching resource. “Tutors” were final year medical students in March 2020 who had just completed finals but were awaiting their results and not yet working as doctors. Tutors produced online teaching material on the RLP. There were no exclusion criteria.

### Recruitment and data collection

After the remote learning teaching had finished, Facebook posts and three emails were sent between 07/03/2021 and 26/04/2021 to Learners and the Tutors. This was purposive sampling. Interested participants contacted researchers using an independent email and were sent a participant information sheet (PIS) and consent form. After consent was obtained, participants were sent a demographics questionnaire (Additional file 1: Appendix 1) and zoom interview invitations. Interviews were conducted and recorded on the Zoom platform and lasted between 15 and 30 min. Interviews were one to one and semi-structured (Additional file 1: Appendix 2). They were conducted by JC, KM and JS who were 3 of the RLP Project Leaders.

### Data analysis

Audio recordings were transcribed by JC. Transcripts were independently analysed after data collection by JC and KM using a thematic analysis [19]. Using Nvivo software, the authors became immersed in the raw data and each sentence or phrase was individually assessed, highlighted, coded and categorised into themes by JC and KM. Codes were created to capture the meaning of phrases in an attempt to index the data. From these codes themes were created that grouped similar codes together. After each analysing author created a list of codes and themes they were reviewed together to produce agreed end themes with relevant codes.

## Results

### Demographic data

#### Themes

Thematic analysis identified 5 themes: education (learning/teaching), productivity, data security, professionalism, and usability of the platform.

Tables 1, 2, 3, 4, 5, 6 and 7 detail some quotes demonstrating the themes in this study.

**Table 1:** Demographic information of study participants

Measure	Number
**Gender**	
Male	3
Female	4
**Age (Years)**	
21–24	5
25–34	2
**Participant type**	
Tutor	4
Learner	3

The total number of participants was 7

#### Education: learning

Table 2

**Table 2:** Education [Learning]

Description	Source	Quotation
Learners found the content useful, focused and enjoyed the peer-to-peer aspect of the resource	Learner 1	“The key advantage really is that it was coming from students”
The ability to ask peers on the comment section of the post was liked	Learner 3	“I saw other people put questions up and then I could see the answer which was useful.” “This was by peers and older peers. It was also just quick access to this information.”
The focused nature of the group was important given the disrupted learning due to the COVID-19 pandemic	Learner 1	“A lot of people have come to the realisation that because they didn’t really learn the last year, and a lot of people didn’t really do the work, so it was definitely helpful”
One useful feature was the ability to learn synchronously and asynchronously	Learner 2	“There was also the choice of seeing it synchronously or asynchronously so having a chance to go back to it especially if it wasn't or didn't fit your timetable you could go back to the slides and stuff.”
The informal nature of Facebook empowered learners to revisit learning resources	Learner 2	“It's less formal so there have been times where I dipped in and I had to have lunch and I just left and then caught up with it…this was less daunting.”
Students have even been using this material in the next academic year, despite formal teaching returning	Learner 3	“I've used these resources this year because even though we have a person teaching it's still invaluable to have older years notes to tell you what you need to learn and to help with specific queries.”

#### Education: teaching

Table 3

**Table 3:** Education [Teaching]

Description	Source	Quotation
One tutor felt Facebook made online teaching feel more personal	Tutor 1	“And you can see quite clearly who is in that group by kind of having a look at their Facebook profile and you know exactly who they are.”
Tutors aware that online learning may be less interactive when compared to face-to-face teaching and attempted to make their material more interactive	Tutor 3	“I tried to make it perhaps a little bit more interactive…you had to kind of think about pre-recorded material as well and recording the teachings”
Tutors liked the ability to immediately answer learners’ queries on the comments section of each post	Tutor 1	“it was quite good to have the comment section” "obviously if one person asks a question, then everyone else can read off there and they can kind of talk to themselves so it promotes group learning.”

#### Productivity

Table 4

**Table 4:** Productivity

Description	Source	Quotation
A perceived negative of having a learning platform on social media was ease of distraction	Tutor 3	“I was getting distracted by the messages I was getting on Facebook, which comes through on your chat box function.”
The ability to have notifications straight to their phone, without logging in was also a benefit	Learner 3	“In a way it made me more productive because I was already going to be scrolling through Facebook but now I'm scrolling through looking at learning resources.”
Some users of the platform had previously closed Facebook when working and didn’t like learning resources on the platform	Tutor 3	“I tend not to go on social media whilst I'm trying to do work, um, because of its distracting nature. Um, I think that was kind of the weaknesses.”

#### Data security

Table 5

**Table 5:** Data security

Description	Source	Quotation
Tutors felt that online teaching, and specifically through a forum such as Facebook, made them change their teaching style and made them more cautious of what to say	Tutor 4	“Um, I guess Yeah, it does put you under a bit more pressure because you have to ensure that everything is done, done a bit more professionally”
When tutors were asked if their material was shared beyond the intended audience, this was accepted and a positive aspect of the material being on Facebook	Tutor 3	“I don't think I was concerned about it too. I think it's quite a good thing.”
The public nature of Facebook had unintended consequences. Despite being a closed group, one student tutor was contacted by a doctor who wanted to use their material for teaching	Tutor 4	“I saw your videos. I thought it was a really good” “do you mind if I use this, um, and I’ll credit you””

#### Professionalism

Table 6

**Table 6:** Professionalism

Description	Source	Quotation
When the tutors were asked if medical school teaching should be on Facebook, most thought this was not appropriate	Tutor 1	“I think it needs to be able to be accessed by everyone” ”I don't think that's really fair.”
The mix of professional and social was rejected by the Tutors	Tutor 4	“sometimes Facebook is used against, or social media in general is used against them and say, like the med bikini.” “I don't feel it's a professional platform for an institution to use”

#### Usability of the platform

Table 7

**Table 7:** Usability of the platform

Description	Source	Quotation
Most learners interacted with the material on their laptops, however, one learner used their phone	Learner 3	“Yeah, it's flexible to learn on your phone because you can do this on the go and you can read the question and comments very easily.”
The familiarity of Facebook as a platform enabled learners and tutors to find materials easily	Learner 2	“I feel like I've been on Facebook for years and it kind of becomes second nature after a while
What is it about Facebook which makes it easy to use that could be translatable to other online learning platforms?	Tutor 2	“the main drawback I found with other platforms was having to click through lots of links, the information not always being clearly available. I think that problem was eliminated a bit by using something like Facebook”
Learners felt the structure of these modules were well organised because students led the project	Learner 2	“modules were categorised, the different modules and everything and even just having the titles helps a lot because it was very relevant to my learning”
One tutor commented on the ability to edit, delete, and post their own material on the platform made them feel more in control	Tutor 4	“We're also able to delete it. Whereas things like Blackboard, you would have to submit to someone getting those files across and then not necessarily being able to take them down” “It gave you a lot more sort of autonomy as, as the educator”

## Discussion

When additional resources are provided for students, these are often received positively. The novelty within this study pertains to the teaching platform Facebook. Overall, Facebook received a positive reception from both learners and tutors. Within our data set, key themes emerged to help us better understand the utility of Facebook and what makes a good online peer-to-peer learning platform.

### Learning

Learners enjoyed peer-to-peer learning, which has been shown to be an effective teaching method [16], and felt they received more focused materials when compared to traditional lectures. This same efficiency of learning was reported in a 2015 qualitative study which remarked that Facebook peer learning was useful for sharing “Tips and tricks” [5]. However, it must be mentioned that peer learning delivers the content students want and not always the content that faculty at the university think students need. Peer-to-peer learning utilises constructivist principles, by helping learners build new knowledge based on their old knowledge. Learners recognised this constructivist process occurring; “they (tutors) would try to relate it to this is what you need to know…” [Learner 1]. As the tutors are student peers and have already recently constructed new knowledge, they are in a good position to help facilitate the same process in learners; “…having complex topics explained by someone else who's gone through the resources recently and learnt it last year…” [Learner 3].

Tutors and learners also remarked that the comments section on Facebook facilitated group discussion that can benefit both parties. A systematic review in 2013 specifically highlighted that participation in course blogs or comments section improved grades and was a key benefit of SoMe use in medical education [20]. Indeed, students who interact more with SoMe comments sections tend to perform better in objective assessments [10] and feel more comfortable asking questions in a forum when compared to ward rounds and clinics [21]. The learners education was facilitated by social interaction and context, as depicted by social learning theory and connectivism. These are fundamental theories underpinning learning on SoMe [22]. As Facebook is an online social platform, it is well designed for communication. Both learners and tutors can interact in the social environment, demonstrated through use of the comments section. Underlying this is a community of practice that is online [23]. Tutors and learners are from the same medical school, bound by common goals and interests and are collectively going through a learning experience to motivate each other and reinforce learning via social mechanisms. Connectivism allows for formation of digital networks to challenge students whilst educators facilitate the process [24].

Another benefit of remote learning as an online platform was the ability to interact with the materials both synchronously and asynchronously. Particularly with how the COVID-19 pandemic has disrupted education, it’s clear the way students engage with materials will change. The ability for students to access these resources as they wish allows optimal learning tailored to the learners individual schedule and this benefit has been reflected in the literature during the pandemic [25].

### Teaching

An overarching theme of the teacher-learning environment through this platform was that it was easier to communicate when compared to traditional online learning. The ability for tutors and learners to know who they’re talking to via Facebook profiles breaks down traditional hierarchies and makes it easier to communicate [26]. In this study, the fact that the learners and tutors were students in the same medical school may go some way to explaining this lack of hierarchy. Personal status may be more explicit to see on Facebook than when teaching face-to-face, but as more educational platforms move online, or move specifically to SoMe, this may be an area for further research. Interestingly, one paper looking at workplaces during the pandemic found that in video conferencing some felt traditional hierarchies were lessened due to seeing children, pets and homes in the background of video calls [27]. It may be that having a personal Facebook profile has a similar effect. Another benefit of Facebook over traditional teaching programs is the cost. For lower resourced educational institutions, SoMe platforms may be a credible alternative to the traditional online learning management systems higher educational institutions use. An additional observation was the variety of online material developed by the tutors on the platform. It included polls, online assessments, screencasts and Q&A’s. It was, in many ways, far more varied that traditional face-to-face university teaching and catered for different learning needs in a synchronous and asynchronous fashion. This links back to an overall ongoing discussion about the future of higher education learning, and the role of online teaching [28]. But also highlights that peer-to-peer teachers recognise the importance of varied teaching in a curriculum and felt that the Facebook SoMe platform facilitated them to deliver these different modalities of teaching. D’souza et al. describes in detail the applications of SoMe in medical education from peer-to-peer learning to ‘tweetorials’ and Instagram videos. Higher education institutions need to grasp this as a potential platform for learning.

### Productivity

Facebook is traditionally seen as a platform that wastes your time, not just for students but also for tutors and doctors. In a recent study, one doctor wrote “you can spend hours and hours just scrolling going from Twitter to Facebook to Linkedin” [29]. It’s clear Facebook also has the capacity to distract in lecture theatres too [14]. In our data, a common theme emerging from both learners and facilitators was that a learning resource on Facebook made it easy to scroll. The extent of which this distraction leads to poorer grades remains subject to debate but there are studies suggesting that being distracted and multitasking on SoMe may lead to poorer memory recall and cognitive performance [30, 31]. With around a third of the world population using Facebook [32], it’s not unsurprising that both learners and tutors enjoyed the familiarity with the platform. This point is particularly important given the remote learning platform was designed and the Remote Learning Project began delivery within a matter of weeks. Learners and tutors did not need to learn how to use the platform, supporting a quick delivery. They were also able to use the platform on different devices (such as phone, tablet or laptop), allowing flexibility of learning in different locations. One learner suggested the intuitive module design could have been because the tutors were themselves senior students who were structuring the content. This does highlight the importance of students being a part of curriculum and learning delivery. During the pandemic, higher education moved almost all teaching online and there is now a very open discussion about how much of undergraduate teaching should be online vs face-to-face, with universities often adopting a hybrid approach [33]. This forms part of a wider vision of a university smart campus, allowing education to be better accessed by learners on different technology platforms [34].

### Data security and professionalism

Within medical education, SoMe is most easily described as a double-edged sword, particularly with regards to professionalism [24]. The tutors enjoyed the possibility that their work may have a larger audience than first intended. It’s also clear that when making these resources the tutors potentially developed higher quality resources, knowing it was online. Generally, the literature suggests that students are comfortable about using SoMe with colleagues for sharing resources and reflection [35]. Despite this, one major barrier of using Facebook for education would be student perceptions of SoMe and professional life. It’s generally considered that most SoMe platforms, but in particular Facebook, are seen as a personal and not professional space – particularly when compared against other platforms such as Linkedin [36]. Students are aware of previous SoMe controversies such as the Med Bikini fiasco and the redacted paper by Hardouin et al., 2020 [37]. With papers evaluating how much publicly available information is on SoMe profiles, it’s not unsurprising students may not like this dichotomy [38, 39]. As a result of the discourse between SoMe and learning platforms there has been a recent call to develop guidelines for professional conduct, particularly for use in medical education. Educational models such as connectivism suggest SoMe is going to become an ever important feature of modern learning [24]. The choice of learning platform, in most cases, is made by the tutors. It’s clear looking at both learner and tutor perceptions that learners had no issue with Facebook as a learning platform, but tutors did have significant reservations. This may go some way in explaining why educational establishments have pushback to learning platforms being on SoMe.

### Strengths & limitations

The Remote Learning project used Facebook. This was a novel concept for the medical school. The Remote Learning project involved 52 tutors and approximately 510 students from years 2–4 of the course.

The study to investigate the users’ views of the learning platform took place approximately 1 year after the RLP teaching had finished. The data for analysis was collected from volunteers who had taken part in the RLP. The response rate to take part in the study was low. The data was gathered from online semi-structured interviews that have credibility but allowed flexibility for the researchers to explore experiences within the interview. Both tutors and students gained different experiences, and these were interpreted using thematic analysis, allowing the researchers to generate themes.

A limitation was that the semi-structured interviews were conducted online. It is not clear yet in the literature how this changes the outcomes of interviewed qualitative data when compared to a face to face interview and is a potential area for further research [40]. The interviewers were also project leaders and had worked as tutors, therefore their own experiences will have influenced questions asked in the interview and the interpretation of the responses.

In an attempt to enable the study participants to give their own opinions, it was important we allowed time for interviewees to speak freely and follow up questions were asked to explore unanticipated answers. The experiences of the interviewers may have also enriched data collection by being relatable to the participants. The findings are transferable to other courses who may wish to use SoMe in medical education. Overall, it is clear there is a need for more evidenced based research in the use of SoMe in medical education.

## Conclusion

This study revealed that Facebook was a suitable medium for delivery of peer-to-peer teaching with advantages over other learning platforms. Facebook is a free alternative to traditional learning platforms and students found it familiar, practical, and easy to use. Both students and tutors felt the personal nature of Facebook had the capacity to break down hierarchies. Some students were concerned that Facebook crossed both personal and professional boundaries. Most limitations of Facebook came from tutors; data security, professionalism and the possibility of student distraction. Educators and faculty wishing to deliver online teaching both on Facebook and other online platforms should consider developing training and guidelines to better support and guide educators on the effective and safe use of SoMe as a learning tool.

Future research could focus on exploring other SoMe platforms. In addition, if possible, higher levels of Kirkpatrick’s model moving from measuring the reaction of learners to evaluating if this online learning method has any impact on examination performance. Further qualitative studies could adopt a grounded theory approach generating richer and more reliable data to help better understand the themes identified in this study.

## Supplementary Information

**Additional file 1: Appendix 1.** Online Pre-questionnaire. **Appendix 2.** Semi-structured questions for learners.

**Additional file 2. **Raw Data.

## Data Availability

See supplementary file for the raw data used in this study.
