# Supplementary material for: Online medical education using a Facebook peer-to-peer learning platform during the COVID-19 pandemic: a qualitative study exploring learner and tutor acceptability of Facebook as a learning platform
Source: BMC Med Educ. 2023 May 1;23:293. doi: 10.1186/s12909-023-04268-3 (PMC10150675; doi:10.1186/s12909-023-04268-3)
Supplement: Supplementary file 1 — Additional file 1: Appendix 1. Online Pre-questionnaire. Appendix 2. Semi-structured questions for learners. [file 12909_2023_4268_MOESM1_ESM.docx]

**Appendices:**

**Appendix 1**

***Online Pre-questionnaire***

Age: 16-20 21-24 25-34 35-44 45-54 55-64

Gender: Male Female Other Prefer not to say

Tutor/learner

**Appendix 2**

***Semi-structured questions for learners:***

1. On average how many hours per week did you spend using remote learning?
2. On average how many hours do you usually spend using Facebook?
3. How did you find the navigation of the Facebook page?
4. How did you find the remote learning page on Facebook?
5. Did you find the Facebook page familiar?
6. What were the benefits of using Facebook?
7. What were the limitations of using Facebook?
8. What are your thoughts on the medical school using Facebook in the future as a learning platform?
9. Did you find yourself distracted by social media due to the learning resources being on Facebook?

***Semi-structured questions for tutors:***

1. How did you find Facebook as a teaching platform?
2. What were the benefits of using Facebook?
3. What were the limitations of using Facebook?
4. What are your thoughts on the medical school using Facebook in the future as a learning platform?
5. Did you find yourself distracted by social media due to the learning resources being on Facebook?
6. Did you do anything different when developing teaching materials for Facebook when developing other teaching materials in the past?
7. Were you concerned that your teaching materials were being shared more widely than you would like when being posted on social media?
8. Were you concerned that non-UEA students would access your data?
