# Supplementary material for: Online medical education using a Facebook peer-to-peer learning platform during the COVID-19 pandemic: a qualitative study exploring learner and tutor acceptability of Facebook as a learning platform
Source: BMC Med Educ. 2023 May 1;23:293. doi: 10.1186/s12909-023-04268-3 (PMC10150675; doi:10.1186/s12909-023-04268-3)
Supplement: Supplementary file 2 — Additional file 2. Raw Data. [file 12909_2023_4268_MOESM2_ESM.docx]

**RAW DATA**

**Tutor 1:**

Researcher: how did you find Facebook as a teaching platform? Um, yeah, it was fine.

It was useful, it was easy to use and quite self-explanatory. it just kind of did exactly what you wanted it to do.

Researcher: What were the benefits of using Facebook?

Tutor: Um, it was simple. I was used to the user face because obviously I use Facebook quite a lot. Um, and, um, it was easy to use.

Researcher: What were the limitations of using Facebook?

Tutor: I don't think everyone, he, um, I don't think everyone has access to Facebook. So I think obviously it's initially like a social platform. Um, and then if it's used to things like teaching, then it can be limited for people who don't have like a Facebook profile. And then for that, they have to get one to access teaching.

Researcher: What are your thoughts on the medical school using Facebook in the future as a learning platform?

Tutor: So I think, like I said, like, I think it needs to be able to be accessed by everyone. And especially nowadays, I think there's a lot of controversy over social media, especially in our profession as doctors and stuff. So, if people don't have Facebook because they feel like they don't want to expose themselves to like that kind of social platform. But then they're going to be limited if they don't get access to teaching, then I don't think that's really fair.

Researcher: How do you mean like with the social media?

Tutor: So then like, obviously, like Facebook is one of the big social media platforms. And I think we're taught, especially from our medical school in particular, to be careful on these kind of, um, social platforms. Um, because it can have repercussions on our careers, especially if we're posting kind of like inappropriate things or. Yeah. Um, so therefore I think some people feel like they don't want it because they don't want to take that risk. But then if they're going to be disadvantaged by not getting access to learning. And then I think that's quite tricky and people shouldn't be forced to get it because some people genuinely just don't like it, because they don't like the idea of social media. Um, they, they shouldn't be, it shouldn't be. Um, I think things like zoom and other things like teams, which are a bit more professional and purely just for kind of work, um, sometimes kind of benefit over Facebook.

Researcher: Did you find yourself distracted by social media due to the learning resources being on Facebook?

Tutor: Oh yeah. Yeah, definitely. I think, yeah, it's just like there. So you're on the platform anyway. So then it's just so easy to like, have a scroll, um, when you're like kind of dipping in out. Yeah.

Researcher: Did you do anything different when developing teaching materials for Facebook when developing other teaching materials in the past?

Um, not really. No, not in particular. Um, uh, probably was a bit more careful on my, you know, the notes section where you, um, where you sometimes might discuss cases and stuff. I like really made sure that there was absolutely no, like, um, not that I would anyway, but like no identifiable kind of information, but not, no, I don't think so.

Researcher: Were you concerned your teaching materials were being shared more widely than you would have liked when being posted on social.

Tutor: Um, no, cause obviously we used what we used. We used the big groups didn't we? Um, so actually there were quite a lot of people in those groups, but actually it probably was a bit nicer. Cause I feel like sometimes. Send them by email, you don't always get the right emails and stuff. So people self joining those groups or being shown how to join those groups is actually easier to share things quicker.

And you can see quite clearly who is in that group by kind of having a look at their Facebook profile and you know exactly who they are. Um, actually it was probably a bit, probably is actually a bit nicer.

Researcher: Were you concerned that non-medical students would access your data?

Tutor: I mean, not really. I mean, the teaching wasn't, I never really thought of it like that. I remember it was private though. Um, I, it was never a concern for me, I suppose it could be a concern. Um, but the stuff that I was teaching wasn't really like, I don't really care about. Random people wanted to learn from it. But I suppose I know, I suppose it was private and the admin like accepted people. So no. say it was private.

Researcher: Any further things you'd like to add about Facebook as a learning platform?

Tutor: Um, it was quite nice to have the different groups. So I remember we did it kind of weekly? So that was like, Specific modules and then specific weeks and then also it was quite good to have the comment section. Cause obviously if you're sharing like a, um, Like a slide show via email, you might get seven different people asking you the same questions, whereas when they post it on the platform underneath where you posted your presentation, um, obviously if one person asks a question, then everyone else can read off there and they can kind of talk to themselves so it promotes group learning. Whereas if you do it via email, they'll all just email you. Um, so I think that was actually quite good. Um,

Yeah, obviously it's a bit difficult with the, um, If you, if you feel like your Facebook is more like less professional, and then obviously your wanting to go on the site, learning platform with kind of people like tutors and stuff. And as you on your profile picture like drinking beer or whatever, then it's not, I don't know. I do think there’s a cross over between the two and some people will want them separate. You know.

**Tutor 2:**

Researcher: Um, so the first question is, how did you find Facebook as a teaching platform?

Tutor: I thought on the whole, it was very good. I thought it allows lots of information to be shared with the target audience, um, you know, on a week by week sort of basis as well. So, you know, more and more would be gradually be, be added. Um, it was very convenient. Um, you know, it's easy to use. Most people I'm generalizing, but most people are able to use it, and have access. Um, and yeah. Yeah. I, I think on the hole it’s very good.

Researcher: Great. Um, so you touched on a few points, um, but could you just elaborate a little bit more, what were the main benefits of using Facebook as a teaching platform during the remote learning?

Tutor: Sure. So I would say the main benefits were ease of access. So it's a single platform. You don't have to go through, you know, lots of different links. Everything was there almost always on one page. Um, so I think, you know, although I didn't access it for my own learning and produced some of the content, I think it would be very easy for students to access, um, uh, sort of touching on that as well. It's how easily the information is disseminated because, um, you know most people have access to Facebook.

Tutor: Um, and you know, I think there was a high uptake across the medical students. Um, you know, joining the groups that you guys had set up, um, to help with their learning. So I think those were the two main things for me.

Researcher: Great. That's really useful points. That's on me. Um, what were the limitations of using Facebook?

Tutor: Yeah, so I would say. I would say there were fairly few limitations actually. Um, yeah, I, I suppose one is being, being distracted is one I would envisage, um, you know, again, the, that, that troubled me less putting the content on there. Imagining that for the medical students possibly, but from my own personal perspective, not, I don't really see any limitations with using Facebook's a platform.

Researcher: Okay, great. And what were your thoughts on the medical school using Facebook in the future as a learning platform?

Tutor: Uh, yeah. I, I, I don't see any downsides. I think it could be used either, Well, perhaps not independently. I remember using things like Blackboard, which I found reasonably good, but the main drawback I found with other platforms was having to click through lots of links, the information, not always being clearly available. I think that problem was eliminated a bit by using something like Facebook, um, where the information's there a lot quicker. So I think that saves time. Um, and I think the medical school using that would be a good thing. So that, that could supplement where, where they store all of the different presentations. Um, yeah, it might be a good supplementary tool maybe.

Researcher: Okay. Okay. Um, and did you find yourself distracted by social media, due to the learning resources being on Facebook?

Tutor: Did I find it distracting? Um, no, not, not myself. Um, the, as I mentioned, I can imagine it, it could be, but if you are, I suppose, going on Facebook for the express purpose of accessing the information, then perhaps you would be less likely to get distracted. So I think from my perspective, the jury would still be out. But Um, I can't see that being a problem either.

Researcher: Okay. And, um, did you do anything different when developing teaching materials for Facebook compared to when developing teaching materials in the past?

Tutor: Uh, yeah. I wou, I would say so. I think when I've, I've made presentations before and Uh, perhaps, you know, in, in the presenter cues bit, I I've given myself, you know, some, some cues for things I would do for a verbal presentation when presenting that information, you don't have that luxury, you know, during a pandemic or when you're delivering the teaching on Facebook. So, something I didn't do, but would do in the future when developing resources would be things like audio. Um, you know, overlay, which I saw other people doing. And I think, you know, that's definitely good. Um, in terms of other things doing differently, um, not, not lots. I think it's very similar, you know, if you're preparing PowerPoints, you just, um, Yeah. I, I don't think it differed greatly from, from previous preparation of presentations there, but there's just other other factors to consider because, you know, you're, you're sort of limited with what you can do.

Researcher: Okay. And were you concerned that your teaching materials were being shared more widely than you would like when posting on social media, such as Facebook?

Tutor: No, I, as I recall, they were closed groups on Facebook. So that, that eliminates some of, some of that. Um, you know, the medical students could share them with other people. I personally wouldn't be bothered by it by that. Um, you know, I think it's good to share resources. They obviously weren't for profit or anything like that. So, so it didn't, it didn't really matter. And I don't think that was an issue.

Researcher: Okay. And the final question is kind of similar, but were you concerned that non new age students would access your data?

I wasn't concerned whether I thought it was happening. I don't know. Again, I, I didn't see that personally. Isn't it?

**Tutor 3:**

Researcher : Okay, thanks for agreeing to be interviewed, Tutor. Um, so the first question, how did you find teaching? Um, for, so how did you find Facebook as a teaching platform?

Tutor: Um, I thought it was um actually quite good overall. I feel it was quite easily accessible. Most people have a Facebook account, so a lot of people were able to join the teaching. Um, so it was good in that sense. Um, I also found that, you know, it was easy to use the chat function. Because I have been using Facebook for some time. It was quite easy to maneuver myself around it, um, and know how to organize the teaching and how to kind of upload at the end as well. So I found the whole experience quite good.

Researcher : Okay, great. Great. And so you did allude to some of those, um, points. So what did you think were the benefits of using Facebook?

Tutor: Definitely, um, the audience, you were able to get to a lot of people, um, and because of how easily accessible it is with everyone, um, a lot of people also joined with facebook, you get notifications before teaching, if someone's interested in an event, you'll get a notification about that. So I feel like it got out to lots of people, So that's definitely point number one, number two, the accessibility of it. Was also really, really good. Um, what else? Um, I would say about Facebook's benefits. Um, nothing else is really coming to mind at this time, those couple of points.

Researcher : Okay, great. Um, and what were the limitations of using facebook?

Tutor: Um, because I guess it's like social media platform. Um, I feel like when I was listening to other people's teachings, I was getting distracted by the messages I was getting on Facebook, which comes through on your chat box function. So, I feel like that was a little bit distracting.

Tutor: Um, you can obviously try and ignore the notifications that come in, but because it's just coming up and down on the screen is a little bit distracting. Um, the other thing with Facebook, you know, you get a notification every time someone posts something as well. So that comes up and again, it's distracting.

Tutor: Um, Social media aspect, then it's quite difficult to know where the balance lies between work and kind of, you know, your social media side of things. And I tend not to go on social media whilst I'm trying to do work, um, because of its distracting nature. Um, I think that was kind of the weak, weaknesses.

Tutor: Also. I feel. Even if people are on social media all the time, I don't think people actually go on Facebook to look at lectures and things like that. So they wouldn't really think to go on there to find the resources. Yeah. That's what I think anyway.

Researcher : Okay, great. Um, and what were your thoughts on the medical school using Facebook in the future as a learning platform?

Tutor: Um, that's a really good question. I feel like the medical school at the moment is, um, Blackboard and they keep everything quite separate from Facebook, but saying that then we also have Medsoc who will promote things on Facebook, but that's quite a social aspect, um, of everything. I think to be honest it is a good idea.

Tutor: Overall the ease of using it, the amount of people that you'd be able to access, it is quite good. However, I feel like new platforms such as Microsoft team and zoom have also come into play with COVID going on. And I feel like people have become quite used to using those as well. Um, so I guess if, you know, you want to keep it professional and not get distracted with social media, etc, maybe perhaps look into using those resources because I don't feel Blackboard is as easy to use zoom in Microsoft teams.

Tutor: Um, overall I think probably not to use Facebook as a platform for medical school.

Researcher : Okay, great. Um, um, did you find yourself being distracted by social media? Um, due to the learning resources being on Facebook.

Tutor: Yeah, definitely. I did. Um, I don't know what way there would be to kind of combat the notifications coming through on that.

Tutor: Um, but at the same time, like, you have the willpower not to go on your phone whilst you're in a lecture and stuff like. Do this same thing and just trying to ignore those things. But I think it's a bit of a distraction for those that get easily distracted.

Researcher : Yeah. Okay. Um, and did you do anything different when developing teaching materials for Facebook, um, compared to when you've developed, uh, teaching materials in the past?

Tutor: I think with it being online, Um, as opposed to in person, I felt that not as many people feel competent to answer it online, as opposed to being in a lecture theatre. I tried to make it perhaps a little bit more interactive. Um, and also I felt that, um, having it Online, you had to kind of think about pre-recorded material as well and recording the teachings, which I've never really done before. So that was something I had to consider. Um, and then again, having to upload it all at the end as well. Not something I've done before.

Researcher : Yeah. Okay. Um, and were you concerned that your teaching materials were being shared more widely than you would like when you were posting your teaching on facebook?

Tutor: Um, I definitely need that. It was being pasted fed, and then, um, probably the UEA scope. Um, but I don't think I was concerned about it too. I think it's quite a good thing. Um, in my opinion, and I wouldn't have minded, uh, you know, keen to benefit of it from that.

Researcher : Yeah. Okay. This is almost a related question then. So were you concerned that non UEA students would access your data?

Tutor: No, because at the end of the day, like the slides and stuff that I would've made would had like their references and there would have been able to find that information elsewhere. I don't know. I don't think it's a concern. Um, the only thing I would be a bit iffy about is having my face on it, but again, like it's something educational and I think people would benefit from it. And I think it's a good thing. So. We should, um, do it, but do it not over Facebook, I think, do it over Other platoforms.

Researcher : Okay, brilliant. Um, and finally, so, um, is there anything else that you wanted to, um, mention with regards to your perception of using Facebook as a teaching platform, um, to conclude the interview.

Tutor: I, I think it's, um, it's definitely an option to consider, but I think we've got other platforms coming in that can also be quite useful and quite easy to access. Um, so although Facebook's really good, I think perhaps consider some others.

Researcher : Okay. Well thank you. Um, for letting us interview you, we really appreciate it.

**Tutor 4:**

Researcher : Uh, so how did you find the Facebook, um, remote learning, uh, platform? How did you find it as a teaching platform?

Tutor: Um, so I found the groups really good to split down the modules and the weeks, and it gave each person, you know, responsibility of their own week to cover and where to put it.

Tutor: It was very easy to find and to join. Um, The only negative thing that I found about Facebook is that, um, you, well, first of all, you’re either on Facebook or not so if somebody is not on Facebook, they can't access it. And it also downgrades a lot of the quality. Um, so I was doing like video screencasts of it and so we'd uploads the video, but it'd be in like, say 360. Um, and we'd also have to provide a link to a Google drive. It was a bit more faf to get a higher quality and wanted to ensure that they're getting it at the quality that it was intended. But overall, it's a great platform to, you know, convey the education and people get notifications. And it's a good way to keep up beyond just the thing that you're teaching. So you can do questions, polls, ask people informally, what sort of things they would like to be covered. So, yeah. All, all in all, I couldn't think of a better way of doing it. Mm.

Researcher : So what do you think was the, the Tutorefits of using Facebook over another platform, such as Blackboard or any other platform you use?

Tutor: So with Facebook, there's sort of us as educators, we're very familiar and the like tutees are very familiar with how it works and how to upload, how to use it, where to find work. We're also able to delete it. Whereas things like Blackboard, you would have to submit to someone getting those files across and then not necessarily being able to take them down or edit them.

Tutor: Um, It gave you a lot more sort of autonomy as, as the educator to be able to convey that and puts it in a really clear place rather than getting lost in the folders within Blackboard, which is quite hard place to navigate anyway. Mm Hmm.

Researcher : And what would you say the limitations are of Facebook over other platforms?

Tutor: Um, so I guess with, with Facebook, as I mentioned, You've got to have a Facebook and a Facebook account. And some people have decided more recently to move away from Facebook because of data breaches. And especially like within the medical profession, sometimes Facebook is used against, or social media in general is used against them and say, like the med bikini.

Tutor: And yeah. So, so there’s that side, not knowing whether it's a professional platform to put educational resources, but the Tutorefit is that it's got a large amount of people on it. It's easily accessible, um, and easy to upload or like your, that the stuff that you're trying to try and put across.

Researcher : And what would your thoughts be on the medical school using social media or Facebook as a, uh, uh, teaching platform in the future?

Tutor: Um, [sigh and pause]. Yeah, so… It was good for us as sort of students to get it across to other students. But I don't. Yeah. I don't feel it's a professional platform for an institution to use, um, to teach, um, like during lockdown, I went to a few other, um, like lectures or educational sessions, which were advertised on Facebook, but then say like hosted on zoom.

Tutor: And it was a good way to do that. But as an institution you're paying for it, I wouldn't think that Facebook's their correct medium for like it's professional image to teach on.

Researcher : So why don't you think it's so professional is because you're paying for it and you want something better or is it just because you don't feel it professional enough?

Tutor: Possibly is the, is the payment, you get a free Facebook account? Why would you pay for somebody else to give you the teaching on it, um, there are lots of other websites or courses that they've run on other platforms and you'd be like, oh, why, why, why can't you do something like that? Um, and with the, the privacy in terms of your, your personal data, And the distractions that like it, as soon as you log into Facebook, sometimes you think about doing one thing and just say, I'm just going to look at this message. Cause I need to download that. And then you get sucked into everything. And if that was then your educational platform and you're getting constant notifications and, um, I think that would detract from the educational experience if it was being delivered on Facebook.

Researcher : Yeah. So did you find yourself getting distracted on social media often when you were doing these teaching sessions?

Tutor: Um, mm, I, I didn't, because I would sort of pre-record them and then upload them onto Facebook. However, if I was as a, like an educatee, sort of like, watching these videos that were like ranged to about 20 minutes or so. And then it does like a little cutout, but then you can still see the main border of Facebook around the outside.

Then you can get a notification here and a notification there, then you're oh, I'll reply to that. And then you've forgotten where you got to and maybe you didn’t get all of the Tutorefit of being completely engrossed for that 20 minutes in it.

Researcher : Hmm. Mm. Did you, as a, as an educator, do anything different with your teaching materials? We talked about it a little bit with Facebook, um, in comparison to other teaching sessions you've delivered in the past either face-to-face or over zoom.

Tutor: No, no, not particularly. I saw that I'd done some things different from other people. A few people did like narrated PowerPoints or just PowerPoints with like, um, scripts underneath the, as it did a full recorded video. Um, But it didn't include my face on it, which I have done on like subsequent lectures, um, to, to build that sort of like personal interaction.

Tutor: And I think that was more of a development as me as an educator, rather than not wanting to put my face on it because it's Facebook. I probably could have done that and should have done that. Um, to help people recognize who it is it's talking, but no, I, don't

Researcher : that’s probably a development of us as educators and as learners through the pandemic. I mean online. It's annoying when people don't put their face up and interact with you now, you know, it's different. Isn't it. It's kind of weird. Um, we we’ve touched on it briefly - Were you concerned that your teaching materials were shared more widely than you would like, um, as it was on Facebook? Or do you not mind that?

Tutor: Um, no, like I, I didn't mind it and I didn't really think about it. Um, but then I got an email from a lecturer in Malaysia. Um, I can send it over to you if you want.

Researcher : [Laughs] Okay.

Tutor: I, um, uh, and it said “I saw your videos. I thought it was a really good” and I think it was like a hypercalcemia one. And they're like, “do you mind if I use this, um, and I’ll credit you”

Tutor: Um, and I was like, yeah, go ahead. Here's the link. So you can actually download it rather than just watching it live. And then as well in the email, I said like, how, how did you find out? Because I wasn't sure how this person is, whether it was because, they would have had to join the Facebook group to be able to see it, or maybe.

Tutor: They had a friend or a colleague from, um, but she didn't, she didn't reply to that. Um, so I still have no idea how it got there. Um, but if anything, it was really nice that it was shared a bit more widely and went beyond the sort of like intended audience as long as they got Tutorefit from it.

Um, I guess Yeah, it does put you under a bit more pressure because you have to ensure that everything is done, done a bit more professionally and is completely up-to-date and correct, because it is shared more widely than you intended and you say, say something wrong or, um, something that might be taken out of context even, um, that that could be, yeah. That, that, that sort of thought would be on the back of your mind if it's getting shared wider than you intended it for it to go.

Researcher : Yeah. But you didn't mind that.

Tutor: No, I didn't mind that I found, I actually really enjoyed getting the email.

Researcher : Yeah I’m sure you did.

Tutor: I was like, oh yeah, great. Feel free.

Researcher : Um, Were you concerned that non UEA students would access, access any of these things, given that the, I suppose that the teaching material was specific to your curriculum, but that didn't really matter either by the sounds of things.

Tutor: No, like it was like UEA specific, but medicine is very generic across all the universities. They might cover it at different points and if it got spread wider and they found it useful, I've got no problem with that. And if they didn't find it useful and just didn't message me that they didn't find it useful. That's great as well.

Tutor: But yeah, if anybody beyond the intended audience found it useful, I think that's, that's a good thing.

Researcher That is all the questions, that's it? Yeah. Very sure. Um, is there anything else you wanted to say about it or, uh, is that that?

Tutor: No. No, that that's everything that I wanted to say for it. You've definitely probes, um, some ideas, um, cause I hadn't really considered whether institution could use Facebook for teaching before.

Tutor: Um, Talking off the cuff, but yeah, like, yeah. I don't think it's the right, right place for it, but yeah, food isn't, isn't great either. So, uh, I, it's a, it's an interesting question because yeah, exactly. You're right. Like Blackboard's rubbish and it's very clunky and like, it just does it. It's not really that user-friendly and Facebook's the opposite is very user-friendly.

Tutor: Then obviously you like, like you said, you're distracted and you know, you have this crossover of like teaching and social stuff and whatever. So I don't know what this solution would be bad for being better. Yeah. And thinking about it as well, like say when I get to your stage or like, as soon as I become a doctor, Um, I'm sure.

Tutor: I don't know if you've changed your name or a lot of your colleagues have changed your Facebook names. And then so maybe you wouldn't want your personal profile to be associated and then find everything. Whereas like, as a student myself, like, like the peers that I'm talking to anyway, they're able to see everything. So I don't mind that. Um, but maybe as soon as there's now professional boundary between it. It then becomes less appropriate to use it, or if, even if there was maybe a institutional Facebook account that you could use, that would be better than your own personal one. Um, but then it's a bit more complicated again, you've got all the logins and passwords and reset it and yeah,

**Learner 1:**

Researcher: On average how many hours per week did you use remote learning?

Learner : Ummm, let mke think back. Ummm I probably used it about 5-6 hours as it would encompass all the LO’s and to go through it. At least. So probably about 5-6 hours.

Researcher: So the second question, on average how many hours do you use facebook itself during the week.

Learner : Per week? Ohhh ummm it’s embarrassing to say. Ummm I feel a bit guilty to say. It’s probably about 30 minutes a day but a lot of the things I used for the umm remote learning were done on powerpoint and I’d just go on facebook so I’d probably say I go on facebook about 4-5 hours per week.

Researcher: How did you find the navigation of the facebook page?

Learner : It was easy enough to access ummm yeah and you have the various groups for the module you were on.

Researcher: Did you find you could find the thing you wanted to?

Learner : On the whole yes as you ummm because at the beginning of the week you would find things uploaded and they’d always be the stuff there for the week. Although, for heam/derm week 9 there was one week that was missed. There wasn’t anything uploaded but I think that was more the systemic and rash week. Apart from that that was pretty much it. Every other week had stuff.

Researcher: Umm so how did you find the remote learining page on facebook?

Learner : Ummmm so how did I come across it or my experience using it?

Researcher: Ummm Your general overall experience

Learner : Overall extremely positive, it was a great learning resource. We were very fortunate to get a hand of. Ummm the first wave happened at the time and I felt ummm I felt the content was for more selective and far more focussed which the lectures were, ummm I mean I found the lectures in all honesty quite poor for the module, for dermatology in particular, for heamatology they were good yeah. Ummm it was very focussed, it was to the point. I liked the commentary they would put on. Because they would try to relate it to this is what you need to know, this is what comes up in exams and yeah it just felt it was a lot more focused and it was that much easier. In lectures they tend to bombard you with unessisary information at times and it was often too long. Yeah.

Researcher: How did you first hear of the facebook page?

Learner :

Ummm I think I must have seen someone else using it, who I was friends with, yeah and that sort of comes up and someone must have advertised it I think.

Researcher: Did you find the facebook page familiar?

Learner : Yeah to use, yes. I use facebook and are familiar with facebook groups. Other facebook groups can be bombarded with 1000’s of posts but this one was quite focussed and there was defined content on it.

Researcher: Did the fact you found the page familiar umm help at all in using it or learning from it

Learner : Yeah, I would say so, sort of if you’re looking for an older post you just need to scroll down a little longer. Just little bits like that really.

Researcher: Okay. What were the benefits of using facebook?

Learner : The benefits were, as much as I hate having facebook for various different reasons it’s still really useful, particularly for medicine as, like I said, you know if other people can advertise particular revision events or advertise this remote learning group which I was able to get hold of and have access to which was definitely a huge positive to learning in the current circumstances they were last march.

Researcher: Yeah. You mentioned earlier, comparing them to lectures as well. Did you find there were any benefits using the facebook page over lectures or other styles of learning.

Learner : Yeah 100%. I find with the lectures, and I was saying this to my housemates the other day when we were doing some revision that there’s often far too much cont… the key points will get buried in other unessisary things and so yeah you were able to really focus out the points and say these are the features, these are the investigations, here’s the treatment. It’s nice when there’s a commentary over as it’s good to listen to and listen if there was extra points, but it was kept focussed and to the point which is something that I have found, particually being at medical school for three years now, these lectures are a bit unessierily ummm long as in like 5-6 hours of lectures a day. I mean this last module I have found these lectures pretty umm, well I haven’t found them that useful to be honest.

Researcher: Yeah they can be quite long. Umm were there any other benefits …. That you found using the page?

Learner : Ummm that it was useful as it helped me keep uptodate with the work. A lot of people have come to the realisation that because they didn’t really learn the last year, and a lot of people didn’t really do the work so it was definatly helpful as it had all the work there and it was sort of, as well as the learning objectives so I was able to go through it and transcribe the notes and stuff. Yeah, it helped me keep up with the workload too.

Researcher: Yeah, did you, when you used it, did you use it week by week or did you use it flexibly in terms of your own work you were doing?

Learner : It was mostly week by week because I ummm one thing I like to do now is to try and keep up with the notes and get the notes done for that week because umm, particularly for year 3 content, it’s amazing how much stuff there was. I was trying to mostly keep up week to week and ummm then yeah I did do a small bit of the recorded materials for revision at the end of this year. Get a few more notes to end the year.

Researcher: Umm so, is there anything else you wanted to mention about that or are you happy to move onto the next question?

Learner : Ummm so, what were the limitations of using Facebook? I mean, the only downsides were that umm, particualry for some modules, they tended to do live lectures were as for module 3 it was mostly just ummm pre-recorded or pre-done. That’s the only disadvantage really, it would have been useful to have a round up thing. But really both resources were just as useful.

Researcher: Were any of the live things recorded at all do you know?

Learner : Umm yeah you can re-access them on facebook so that’s an advantage. The only disadvantage was that sometimes they were on the google drive and sometimes they were on facebook so umm accessing them was a little bit of a neusence sometimes.

Researcher: In terms of finding the content?

Learner : Yeah sometimes you would end up looking on the google drive. But it was easy enough to access it. Like I said, apart from one week were there wasn’t anything, maybe week 10 module 3 there was no resources.

Researcher: what was the conetent like throughout the week on facebook, you mentioned some weeks had missing conetent

Learner : Just week 9 and 10 module 3 which was missing. On the whole all the resources were there. The haematology and dermatology stuff was really good. It would have been nice if there had been week 9 and 10 stuff but yeah that’s fine.

Researcher: What are your thoughts on the medical school using facebook in the future? As a learning platform??

Learner: Ummm, I mean the key advantage really is that it was coming from students whereas, well as being a student you know the experiances you are going through, you know what to expect, you know what you need and don’t need to learn, which I thought was a really huge benefit. It certainly was a good site for accessing information, in terms of students accessing it. I’m not sure how much benefit the medical school would get out of using it, because obviously there are some concerns about social media and stuff which I know some, well almost all medical students are aware of. That’s my initial thoughts.

Researcher: In terms of social media then, what are your concerns if the medical school are using the facebook platofmr on social media.

Learner : I suppose it would be that they were able to access profiles and accounts but I mean you run the risk of that by having facebook regardless, there are issues these days. That’s about it really.

Researcher: Did you find yourself distracted by social media by the resources being on facebook?

Learner : Ummm I mean to an extent, but umm I would also blame the current situation in the last, to be honest, in the last sort of 13-14 months one thing I have noticed is that my screen time on my ohone has gone up a lot more and I think just the nature of the fact that it is on social media, you’re more inclided to use the social media things because obviously social media are designed in such a way, they have algorithms to ensure that everything you see is something you like or make your bein tick. That’s the only downside I think, but I would say that is more of a priblem with the pandemic (with screen time) and screen time going up.

Researcher: So obviously facebook being a platofmr on social media, on your screen, how would you describe that in terms of benefits or limitations of that?

Learner : I mean the benefits are that it is straight forward to use and access. It was easy to access the group. It was great they checked first you were a student. So you don’t get spam. The downsides are that you end up using the other facebook features but for me that wasn’t too much of a problem. Ummm I could just access stuff and close facebook after that.

It was a great resource, I have been using it this year for module 7, and another housemate has also used the resroces this year. It would be very useful for new students in year 1 and 2, as the amount of content is so overwhelming. It helps bridge the gap towards what you need to know and starts to develop the clinical thinking and reasoning they like you to have.

**Learner 2:**

Researcher: so the first question is how many hours on average did you use remote learning

Learner I would say like 15 15 hours a rough estimate or something like that

Researcher: on average how many hours do you use using Facebook in a week

Learner and probably like 20 minutes a day

Researcher: how did you find the navigation of the page?

Learner not that complex really it was quite easy to use

Researcher: was there any parts of it you couldn't find anything or any difficulties at all going back to things?

Learner I wouldn't say so I feel like I've been on Facebook for years and it kind of becomes second nature after a while.

Researcher: So you didn’t have to think too much about it?

Learner no not really

Researcher: was there at any times did you have problem accessing anything on Facebook or anything that you couldn't find

Learner no I feel like sometimes when you're putting things on or events on calendar on Facebook it can be hard to figure out where they've gone but that might depend on if you are just clicking interested or or where you're going maybe but that's the only thing that comes into my head

Researcher: So you can remember seeing the event and then have no idea where its gone?

Learner yeah basically yeah

Researcher: how did you find the remote per remote learning page on Facebook

Learner I'm pretty good really yeah it's just it's good to have things like the events and live events going on which are then saved which I feel was pretty good really and then modules were categorised the different modules and everything and even just having the titles helps a lot because it was very relevant to my learning and I feel like if it was done by another uni or actual doctors or consultants or professionals they wouldn't have been able to target this learning material to us that's why it was so good it was so good it's that peer learning aspect of this teaching resource on Facebook.

Researcher: you mentioned at the start where everything was split into modules was there anything else you liked about how it was arranged on Facebook and when you were using it?

Learner I liked that there was also the choice of seeing it secret synchronously or asynchronously so having a chance to go back to it especially if it wasn't or didn't fit your timetable you could go back to the slides and stuff not sure about any other things. I think just having something to look at, the slides were nicer. They were very good to follow through rather than just having someone speak to you.

Researcher: did you tend to use it more synchronously or asynchronously how did you use Facebook with regards to this?

Learner I think I used half and half, maybe a bit more synchronously. But I have gone back to things even just to look at slides rather than the actual talk. So yes more synchronously but I have gone back to see the slides and kind of like skim through rather than listen to all again because the synchronous aspect obviously allows questions and interaction which is actually quite nice to have, asynchronously is more just relating back to things that were important to me.

Researcher: did you find the Facebook page familiar

Learner yes I feel like it was a standard page really. Maybe it was just me who didn't come across it would have been nicer to have more pictures rather than just a lot of videos because because Facebook is picture based and seeing a page without any pictures and hour long videos. It was the only thing that was different from the standard Facebook page I guess.

Researcher: How how does having more pictures help you?

Learner I guess things are coming out of the page a bit more really instead of just having a reminder popping up in the notifications having a picture, even just the most generic picture coming up on my feed - this just grabs my attention more. I guess this is just eye catching.

Researcher: so it helps you feel a bit more engaged with the page you say rather than just checking once or twice a week?

Learner yeah exactly

Researcher: what were the benefits of using Facebook?

Learner you can see other people going to it so this definitely pushed me, a lot of my friends my cosmax have joined these things and this is more motivation for me rather than just getting an email through saying things just like any other email. emails have lots of important things like grades and dates but on Facebook it's more welcomed. It's less formal so there have been times where I dipped in and I had to have lunch and I just left and then caught up with it asynchronously on Facebook. This was less daunting.

Researcher: It's interesting how you can see your friends were interested. Did these more social aspects of the learning platform change the way you were learning at all or the way you interacted or did this help you learn in anyway?

Learner yeah I mean definitely there was some competitiveness friendly competitiveness I guess UEA is a friendly competitiveness for other unis might not be but making that small extra step to match the other people that said they were interested in these revision sessions or whatever and it does also make you feel safer whilst attending it means that you may want or this this changes how much you want to answer questions or ask questions during the sessions I mean half of the people on the groups you'll know of so it's not as scary as asking a question at a conference or a lecture.

Researcher: Yes it can be quite daunting. what were the other good aspects of using Facebook?

Learner not trying to keep track of it as as I well I cheque Facebook on a daily basis and you get reminders which is quite useful.

Researcher: what were the limitations of using Facebook?

Learner I feel like it probably doesn't really reach everyone as I have mates who don't cheque Facebook regularly or at all or someone not even being on the platform so obviously it doesn't reach out to everyone and it's also very younger generation kind of thing so if it was to be brought on for things it wouldn't work for older people maybe. I guess however it did reach a wide audience I imagine I just guess not everyone can be included.

Researcher: did you ever have problems yourself using Facebook?

Learner no it is pretty clear in terms of finding the live sessions. Not really, no.

Researcher: thank you. So what are your thoughts on the medical school using Facebook in the future as a learning platform?

Learner I feel like that would be an interesting one to see. It feels like it doesn't feel formal. it feels more social media young generation and lectures are already struggling quite a bit to figure blackboard out which is what they have been struggling with for years. so I feel like it would be quite a change for lecturers. If it was other students it would probably work and be successful but it might require some students who don't have Facebook to actually make an account and the fact you need an account for it can be quite limiting for people. Obviously for blackboard every student has one because it's paid for. But Facebook is a new platform that Facebook well students and lecturers would have to learn.

Researcher: in terms of the medical school using Facebook would you prefer it to be more student lead like last time all faculty lead. How do you think you'd find that being on Facebook? did you have any thoughts or preference says on students using Facebook for peer teaching or faculta using it for teaching or a combination of both?

Learner I feel like student lead things would probably be more successful, generally even just for lecture based staff faculty lead teaching it would be nicer to have a blended learning approach not just to have a PowerPoint and talking and reading off the PowerPoint quite a lot of lectures are well I'm not sure Facebook could accommodate it accommodate this blended approach it's very good for student lead teaching for peer to peer teaching but you expect faculty teaching to be more interactive. students know how to use Facebook but with staff lead teaching it would be much harder. if you see the anatomy page on blackboard it's full of good stuff quizzes and different resource is which perhaps you can't do it on Facebook. if that makes sense. this is a software limitation of Facebook maybe?

Researcher: did you find yourself distracted by the learning resource as being on Facebook?

Learner no well maybe was looking for it obviously had to access Facebook and search for the page and go on it so maybe in those first few seconds and minutes you find yourself scrolling and then you know an instance and then actually accessing the page but whilst the pages accessed that was it.

**Learner 3:**

Researcher: okay the first question is how many hours per week do you spend on Facebook using remote learning?

I saw other people put questions up and then I could see the answer which was useful. during the module at home? probably like 5 hours a day over that. Learning.

Researcher: on average how many hours do you usually spend on Facebook

Learner on Facebook maybe 2-3 hours a day

Researcher: how did you find the navigation of the Facebook page?

Learner very simple really it was very common sense if that makes sense you just click on what you need. I like that you just get notifications. You don't have to keep on checking it you'll just be told about these resources.

Researcher: was there any point to do struggle to find anything you needed?

Learner no I don't think so

RESEARCHER how did you find the remote learning page?

Learner yeah there was a remote learning page and then there was every specific module page. it was more than module specific pages that I used that gave us access to Google drop docs and PowerPoints and everything which was useful.

RESEARCHER did you use the page just for the model you are currently on or did you use any others?

Learner I used the module specific one in that module but since then I have gone to other modules to request access to Google Docs so that I can access resources. I have used them I've used these resources this year because even though we have a person teaching it's it's still invaluable to have older years notes to tell you what you need to learn and and and to help with specific queries.

RESEARCHER did you find the Facebook page itself familiar to use?

Learner it was familiar as I could very easily use it sort of as any other Facebook page. I didn't think much of how easy this was because we grew up with Facebook it was just like every other page so it didn't hinder anything at all.

RESEARCHER what were the benefits of using Facebook?

Learner access to older years notes and also you can put questions up directly on the resource is I never did this myself but I saw other people put questions up and then I could see the answer which was useful. this was by peers and older peers. It was also just quick access to this information.

RESEARCHER Facebook is a social platform did you find that in terms of learning remotely did this make it more interesting or did it alter the learning in anyway?

Learner it maybe it made it maybe not more interesting it just made it more accessible. it's just having complex topics explained by someone else who's gone through the resources recently and learnt it last year rather than learned it decades ago it's it's it's the same kind of level of knowledge that we need to know and that was really useful. it was easier to learn from because of this.

RESEARCHER were there any other useful aspects to using Facebook?

Learner everyone has it I guess so it's not something you have to download it's already there available to use.

RESEARCHER in terms of interactive in the page did you find or the concept was there that you needed over there any times somethings didn't work for Facebook or did you have any other problems with Facebook?

Learner everything was there that was needed obviously you click the links to go onto Google Docs sometimes and then you had to wait to have access. and this was quick but you had to wait for this.

RESEARCHER what what were the limitations of using Facebook?

Learner having to click on Google Drive and wait for access. I guess the actual resources weren't on Facebook there on Google Docs. I can't think of anything else.

RESEARCHER if you had to click on one thing one day and then come back to another day you were having to click it again how did you find this? how did you find to navigate Facebook?

Learner they were limited post it wasn't bombarded it was quite succinct and summarise posts rather than everyone posting everyday it was like one a week and then question answers and sometimes searching for the page was helpful. it was fine.

RESEARCHER have you ever been able to not access things that you've needed?

Learner no not really sometimes you had to request access. but to be honest I wasn't even on the module at that time sometimes it was when I was going back to the resoureces.

RESEARCHER what are your thoughts on the medical school using Facebook in the future as a learning platform?

Learner yeah it's good we're already on it so it's already set up to use I guess the module specific skirt because then you can choose which ones you want to focus on or ignore I think it's really useful to have access and for more access to peer learning and then specifically saying what you should concentrate on because PAL finishes in year 1 say some more former formal access to older years materials would be good.

RESEARCHER would you have any concerns if the site was run by the medical school in terms of it being on Facebook and not being on any other platforms they can be used?

Learner I guess with it being used on Facebook rather than anything else, if it was on blackboard or any other site that we use we don't get notifications straight to us. so it's something you'd have to go on log on and cheque rather than it just being integrated into your phone and everyday.

RESEARCHER that's interesting you mentioned your phone there. Did you use it on your phone or tablet or was mainly just your laptop out of interest?

Learner I interacted with the Facebook page on my phone but the other resource is on the Facebook site would be via the laptop?

RESEARCHER did you find this flexible for your learning?

Learner yeah it's flexible to learn on your phone because you can do this on the go and you can read the question and comments very easily. I always advise off my laptop so when I was using the form or resource is like PowerPoints I would use my laptop.

RESEARCHER did you find yourself distracted by social media due to the learning resources being on Facebook?

Learner I would say not any difference in normal because Facebook is always open so it doesn't really matter if I'm using it on another site all Facebook itself because Facebook is always in the background and I guess I didn't because I went on for that. In a way it made me more productive because I was already going to be scrolling through Facebook but now I'm scrolling through looking at learning resources. This was beneficial rather than just a lot of junk on your feed.

RESEARCHER did you ever have any thoughts about mixing your education and learning with more your social free time or is that something that doesn't bother you?

Learner no I kind of do on Instagram too you can very easily subscribe to medical pages already that just pop up on your feed and then with medicine you should always be learning so it's good to remind yourself of something. you're already there so it's useful to have something useful going on as well.
